# Toward standardization of GenAI-driven agentic architectures for radio access networks

**DOI:** 10.3389/frai.2025.1621963

**Published:** 2025-07-22

**Authors:** Zeinab Nezami, Syed Ali Raza Zaidi, Maryam Hafeez, Jie Xu, Karim Djemame

**Affiliations:** ^1^School of Electronic and Electrical Engineering, University of Leeds, Leeds, United Kingdom; ^2^School of Computer Science, University of Leeds, Leeds, United Kingdom

**Keywords:** generative artificial intelligence (GenAI), radio access networks (RAN), large language models (LLMs), 6G networks, standardization, Telecom, agentic architecture

## Abstract

The adoption of Generative Artificial Intelligence (GenAI) in Radio Access Networks (RAN) presents new opportunities for automation and intelligence across network operations. GenAI-powered agents, leveraging Large Language Models (LLMs), can enhance planning, execution, and decision-making for orchestration and real-time optimisation of 6G networks. Standardizing the implementation of the Agentic architecture for RAN is now essential to establish a unified framework for RANOps and AgentOps. One of the key challenges is to develop a blueprint that incorporates best practices for memory integration, tool generation, multi-agent orchestration, and performance benchmarking. This study highlights key areas requiring standardization, including agent tool specifications, RAN-specific LLM fine-tuning, validation frameworks, and AI-friendly documentation. We propose a dedicated research initiative on GenAI-for-RAN and GenAI-on-RAN to address these gaps and advance AI-driven network automation.

## 1 Introduction

With each generation of wireless networks, managing their complexity has become increasingly challenging, leading to higher operational expenses (OPEX) and total cost of ownership. To address this, 6G wireless networks envision an AI-native design not only to mitigate complexity but also to enhance performance through AI-driven RAN optimization^1^. The Open RAN (O-RAN) architecture Rouwet (2022) has laid the foundation for embedding AI at multiple avenues within the RAN. With the advent of GenAI, this is evolving into an Agentic architecture capable of leveraging LLMs to automate RAN workflows (SoftBank, 2025).

Considering RAN-related tasks, two key avenues emerge where AI agents can play a critical role: (i) Split architectures, which allow for distributed cognition across the protocol stack through multiple coordinated agents; and (ii) Multi-access Edge Computing (MEC) integration into the RAN, which enables the execution of task-specific agents close to the data source. In parallel, GenAI can be seen as both a workload for RAN and an enabler of RAN. In this context, the distinction between AI-for-RAN—where AI enhances core RAN functionalities such as signal processing, network optimization, and predictive maintenance—and AI-on-RAN—where the RAN infrastructure hosts AI-driven applications such as computer vision or federated learning—is critical. Supporting both paradigms on a unified infrastructure presents significant challenges in maintaining service-level guarantees, particularly given the stringent latency and reliability constraints intrinsic to RAN environments. This dual role of AI highlights the need for intelligent orchestration mechanisms that minimize manual intervention while ensuring workload coexistence and strict performance isolation between AI and RAN functions.

A fundamental challenge in realizing this vision lies in the deployment of agent-based solutions across the RAN protocol stack. These implementations demand rigorous performance benchmarking, automated testing, and standardized definitions for agent attributes and toolchains. Several telecom standardization bodies (Giannopoulos et al., 2022; Alavirad et al., 2023) have already advocated for formalizing agent-based architectures and standardizing associated concepts for RAN. However, much of the discourse to date has been guided by optimistic assumptions—namely, that agents can be deployed arbitrarily within the stack and still operate within acceptable latency bounds. In practice, latency constraints at lower layers, such as the Radio Link Control (RLC) layer, are substantially more stringent than those at the application layer, making this assumption problematic.

Furthermore, tool orchestration capabilities—especially those used by LLM-based agents—are typically evaluated using abstract metrics such as irrelevance, as seen on Berkeley Function Calling Leaderboard (Berkeley Function Calling Leaderboard, 2024). These metrics, however, do not yet reflect performance in latency-sensitive, RAN-specific contexts. Additionally, with the increasing relevance of multimodal LLMs in telecom—due to the heterogeneous nature of RAN data—the integration of time-series measurements, performance logs, and domain-specific knowledge into tool-calling pipelines remains an open research challenge.

In response to these gaps, we propose a dedicated standardization study item on Agentic Architecture for 6G-RAN, currently under preparation for submission to the AI-RAN Alliance (AI-RAN, 2025). In this article, we aim to detail the core focus areas and the methodology we intend to adopt in shaping this initiative. The rest of the paper is organized as follows. Section 2 presents the proposed Agentic Architecture for RANs. Section 3 discusses key dimensions and avenues for standardization of GenAI-driven agents for RAN applications. Finally, Section 4 concludes the paper and outlines future directions toward standardization.

## 2 Agentic architecture for RANs

**Figure 1:**
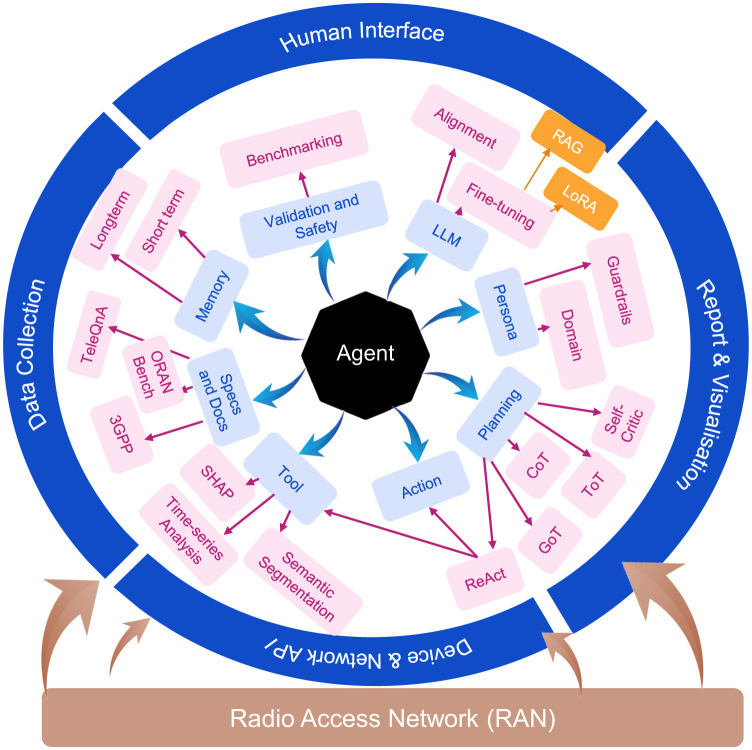
GenAI-based agent architecture leveraging LLMs for automated planning and execution of AI-RAN workloads.

GenAI-based agent implementations exploit the capabilities of LLMs to automate the planning and execution of both RAN and AI workloads. Figure 1 illustrates the proposed GenAI-driven agentic architecture for (RANs), designed to support modular, scalable, and intelligent network management. The architecture is structured around both external interfaces and internal cognitive modules that collectively enable end-to-end automation and adaptive decision-making in RANs. At the outermost layer, the system integrates four key external interfaces. Data Collection Interface includes real-time telemetry, logs, KPIs, and configuration data from RAN components (e.g., RU, DU, CU). This data feeds into the memory and context subsystems for situational awareness. Human Interface module allows human operators or developers to interact with the agents through natural language queries, intent declarations, or manual overrides, supporting explainability and oversight. Device and Network Interface empowers agent to interact with the underlying RAN infrastructure through programmable APIs, such as O-RAN's E2 and A1 interfaces, enabling both observation and control actions on physical or virtualized components. Report and Visualization Layer provides graphical dashboards, monitoring summaries, and structured logs to help users track system behavior, agent decisions, and network status in real time. It supports transparency, debugging, and *post-hoc* analysis. Inside the architecture, the core agentic system—composed of Memory, Planning, Tools, Persona, LLM, Validation and Safety, Specification and Documents, and Action modules—bridges perception and actuation in RAN environments. These components enable agents to reason over complex inputs, plan multi-step actions, and provide transparent outputs through integrated reporting—all in a decentralized, scalable manner suitable for both edge and centralized deployments. The internal modules—memory, planning, and tools—are introduced here and further explored, along with the remaining architectural components in the subsequent section.

While GenAI-driven agentic architectures are gaining momentum in both research and commercial domains (Wu et al., 2025; Wang et al., 2025), their application within the RAN context presents unique challenges that underscore the need for standardized interfaces, behaviors, and performance guarantees. Enabling interoperable and reliable deployment of agent-based systems across multi-vendor and multi-operator environments requires each architectural component to be formally specified and rigorously evaluated through standardized methodologies. In the following sections, we delve into the core building blocks of the proposed architecture.

### 2.1 Memory

This is typically divided into short-term and long-term components, often backed by vector databases (Xie et al., 2023) to support document and embedding retrieval. In general-purpose agents, memory may also store reasoning traces, self-critic logs, and state histories to support planning and reflection. In RAN-specific agents, this component must expand to handle structured time-series data, performance counters, and domain-specific logs. However, no standard exists for representing or exchanging these heterogeneous data types across agent implementations. Further, depending on how the agent is distributed—whether across the protocol stack or the cloud-to-edge continuum—memory allocation and access latencies may vary significantly. This necessitates standardized memory management schemas and access protocols that account for RAN's real-time constraints. Moreover, memory-augmented decision-making in RAN settings requires consistent traceability and auditability, which are yet to be addressed in existing implementations.

### 2.2 Planning

Agents use a variety of planning methodologies, ranging from chain-of-thought prompting (CoT) (Wei et al., 2022) to more sophisticated approaches of ReAct (Yao et al., 2023b), tree-of-thought (ToT) (Yao et al., 2023a), and graph-of-thought (GoT) (Besta et al., 2024). These strategies enable multi-step reasoning, task decomposition, and iterative refinement. However, while these approaches have shown promise in general domains, standard evaluation metrics for planning performance in RAN-specific tasks—such as latency impact, task completion reliability, and error recovery—are lacking. Moreover, agent planning capabilities often include self-reflection and multi-plan selection. Without standardized representations for plan structures, execution traces, and retry logic, it becomes difficult to compare or benchmark different agents under telecom-AI workloads.

### 2.3 Tools

Planning components often culminate in the invocation of external tools—such as network configuration APIs, monitoring scripts, or diagnostic utilities—to complete a task. Modern LLM agents can even generate tools on the fly, using in-context code synthesis. LLM-based code refactoring (Du et al., 2023; Thakur et al., 2023), network configuration generation (Xu et al., 2024; Dzeparoska et al., 2023), and policy translation (Mondal et al., 2023; Wang et al., 2023) are increasingly integrated into telecom use cases. However, without standardized tool registries, interface definitions, or capability descriptors, dynamically generated tools raise significant concerns around security, traceability, and compatibility. For example, the integration of techniques such as Shapley value calculation (Winter, 2002) can help assess the contribution of individual RAN elements (e.g., base stations, cell configurations, user load) toward network KPIs. Yet, the adoption of such tools requires standardized input formats, attribution models, and execution environments. Currently, the use of AI-generated tools in RAN management remains *ad hoc*, and their deployment poses integration risks without consistent operational semantics.

## 3 Discussion

To enable interoperable, scalable, and efficient deployment of agent-based systems in the RAN context, the agent framework must be supported by dedicated standardization efforts. These efforts should address the definition of robust specifications for several critical components and behaviors, including but not limited to.

### 3.1 Tool specification and discovery

Function-level specifications are commonly expressed through standards such as OpenAPI 3.0 (OpenAPI Initiative, 2017), which provide a foundational semantic layer compatible with baseline LLMs. However, these specifications alone are insufficient for building fully functional and interoperable agent ecosystems in the RAN context. To realize a practical agent framework, it is essential to complement such semantic descriptors with concrete implementation guidelines, runtime requirements, and domain-specific execution semantics.

Development platforms such as NVIDIA's Aerial RAN CoLab Over-the-Air (ARC OTA) (NVIDIA, 2024a) and signal processing libraries such as PyAerial (NVIDIA, 2024b) represent critical infrastructure for accelerating the prototyping of agent capabilities. These platforms offer standardized, open-source environments upon which developers can build, test, and validate novel RAN-related agent functionalities. To foster a sustainable and collaborative open-source ecosystem for agentic tools within RAN, we advocate for the creation of standardized guidelines that govern both specification and discovery processes. These guidelines should address not only syntactic compatibility but also execution guarantees, trust, and integration policies.

There are two fundamental dimensions to consider when specifying agentic tools for RAN: (i) Functional Mapping: identifying and mapping key capabilities required across various layers of the RAN protocol stack—ranging from signal processing to configuration and monitoring. (ii) Interaction Scope: defining the scope of agent interactions, including the information elements, service primitives, and control/management entities involved. Any tool integrated into RAN infrastructure must adhere to strict requirements for safety, reusability, and discovery. Without such standardization, siloed or proprietary implementations risk fragmenting the ecosystem and introducing inconsistent or unpredictable behaviors in live workflows. The core standardization requirements for tool on-boarding are summarized in Table 1.

**Table 1:** Key considerations for tool specification and discovery in RAN agentics.

**Category**	**Key considerations**
Reusability and modularity	Design agent capabilities as reusable components; enable plug-and-play integration across systems.
Implementation guidelines and blueprints	Provide best practices, templates, and reference architectures to standardize tool development.
Dynamic tool discovery and integration	Support real-time tool registration, lookup, and composition with evolving AI-native RAN systems.
Safety considerations	Ensure secure interfaces, sandbox third-party tools, and validate all integrations before deployment.

### 3.2 LLM fine-tuning for RAN contexts

Fine-tuning LLMs for RAN-specific agent applications requires standardization to ensure reliability, efficiency, and adaptability. One widely adopted technique is Retrieval-Augmented Generation (RAG) (Lewis et al., 2020), which enhances LLMs with external knowledge sources to improve task-specific performance. Another method, Low-Rank Adaptation (LoRA) (Hu et al., 2021), offers a computationally efficient approach to fine-tuning while preserving performance. An emerging trend in this domain is Telco-RAG (Bornea et al., 2024), an open-source RAG framework tailored to telecommunications standards. While Telco-RAG shows promise, the complexity of 3GPP (3GPP, 2025) and O-RAN (O-RAN Alliance, 2025) specifications presents significant challenges for LLM integration.

There is an urgent need to define best practices for fine-tuning approaches that yield validatable and reproducible results. Currently, validation mechanisms rely on limited datasets such as TeleQnA (Maatouk et al., 2023), which may not fully reflect real-world telecom environments. Developing richer benchmarking datasets is essential for advancing model reliability and domain adaptation. Given the multi-modal nature of telecom data—including structured tables, logs, textual specifications, and performance metrics—there is growing interest in structured methodologies for multi-modal RAG. Two prevailing approaches exist, and selecting the most suitable paradigm for telecom-specific modalities is critical.

- **Cross-modality reasoning with data conversion tools** (Qian et al., 2024): Converts one data modality into a reasoning-compatible format for another.
- **Embodied multi-modal models** (Yang et al., 2024): Models such as PaLM-E (Driess et al., 2023) integrate diverse data types within a unified learning framework.

### 3.3 Benchmarking and validation

The effectiveness and safety of agent-based implementations in RAN are heavily influenced by the underlying foundation models. Variations across LLMs in terms of latency, throughput, task relevance, and energy efficiency (Laskaridis et al., 2024; Nezami et al., 2024) highlight the urgent need for standardized benchmarking and validation frameworks. To ensure fair, reproducible, and comprehensive evaluation, agent performance must be quantified through structured methodologies, as outlined below:

#### 3.3.1 Standardized benchmarking methodology

- Developing a unified evaluation framework that accounts for key performance metrics such as response time, inference efficiency, and task-specific accuracy.
- Defining protocols for testing agent behavior in dynamic telecom environments, considering real-world constraints such as network congestion and resource availability.

#### 3.3.2 Standard benchmarking and validation datasets

- Creating diverse and representative datasets that cover real-world telecom scenarios, moving beyond simplistic datasets (Maatouk et al., 2023).
- Ensuring datasets reflect key challenges in RAN automation, including multi-turn dialogue handling, protocol compliance, and network state prediction.

#### 3.3.3 Safety considerations

- Implementing rigorous security assessments to ensure LLM-based agents do not exhibit unintended or unsafe behavior under adversarial conditions.
- Establishing fail-safe mechanisms to handle incorrect or misleading responses that could disrupt telecom network operations.

By systematically addressing these dimensions, the telecom sector can establish objective performance baselines, fostering transparent comparison between agent implementations. This will enhance the reliability, safety, and interoperability of AI-driven systems across heterogeneous RAN architectures.

### 3.4 Agent-readable specification formats

As 6G-RAN documentation evolves, it is crucial to make specifications accessible not only to human engineers but also to autonomous agents. Current formats are predominantly text-based and optimized for manual interpretation, making them poorly suited for automated reasoning or ingestion (Perez and Vizcaino, 2024). To support agent-based operations in telecom, we propose the standardization of agent-readable specification formats that fulfill the following objectives:

#### 3.4.1 Enable automated ingestion

- Define machine-readable structures (e.g., JSON-LD, RDF, or domain-specific ontologies) (Hu et al., 2024) that allow agents to efficiently parse and interpret RAN specifications.
- Minimize manual effort in processing specifications, accelerating agent deployment and adaptability in dynamic environments.

#### 3.4.2 Support autonomous reasoning

- Develop structured formats that retain contextual and hierarchical relationships, enabling agents to extract actionable logic for tasks like configuration or diagnostics.
- Facilitate knowledge grounding and inference within telecom tasks through logical or graph-based representations.

#### 3.4.3 Ensure standards compatibility

- Ensure backward compatibility with existing 3GPP (3GPP, 2025) and O-RAN (O-RAN Alliance, 2025) standards to promote smooth integration.
- Collaborate with standardization bodies to align machine-interpretable formats with future telecom documentation practices.

#### 3.4.4 Maintain safety and integrity

- Implement safeguards against ambiguity, misinterpretation, or manipulation of machine-readable documentation.
- Ensure version control and digital signatures to validate document authenticity and trace changes over time.

### 3.5 Definition of agent personas and roles

In agent-based systems, a persona defines the agent's role, expertise, and interaction style. While commonly associated with chatbots, recent studies (Mao et al., 2023) show that persona design critically affects orchestration and decision-making in broader applications. Inaccurate or vague personas (Mao et al., 2023) can lead to hallucinations, task misprioritization, or erratic behavior—risks that are unacceptable in RAN, where precision and compliance are vital. To ensure consistency and safety, we propose the standardization of RAN-specific personas along the axes detailed in Box 1.

Box 1 **Agent Personas and Roles**

3.5.1 Role-Based Persona Definitions

3.5.1.1 Predefine personas aligned with specific RAN tasks (e.g., optimization, fault management, configuration).

3.5.1.2 Ensure role descriptions comply with 3GPP (3GPP, 2025) and O-RAN (O-RAN Alliance, 2025) specifications.

3.5.2 Controlled Adaptation via Guardrails

3.5.2.1 Enable scenario-specific tuning while enforcing constraints to avoid hallucinations or errant actions.

3.5.2.2 Validate persona behavior against expected task outcomes.

3.5.3 Safety and Integrity

3.5.3.1 Restrict overly permissive roles that could introduce instability or unauthorized actions.

3.5.3.2 Secure persona definitions against tampering or misuse.

### 3.6 Agent deployment for edge-aware RAN applications

As next-generation web and handset-based applications evolve, agent-based intelligence will become central to enhancing automation, personalization, and network interaction (Kan et al., 2024). These agents will increasingly manage RAN resources, optimize user experience, and support application logic at the edge. Efficient deployment of such agents hinges on managing computational loads through edge offloading and caching (Zou et al., 2023), particularly given the demands of models like RAG. We propose an investigation into the following key areas to facilitate agent implementation on RAN:

#### 3.6.1 Edge offloading for RAG-based agents

- Design frameworks to offload RAG-related computation from centralized systems to edge nodes.
- Leverage RAN's distributed architecture to support real-time, context-aware agent execution closer to end users.

#### 3.6.2 Caching for lightweight inference

- Develop intelligent caching strategies to store frequently accessed models and data at the edge, reducing redundancy and computational overhead.
- Ensure consistency and synchronization of cached content across distributed edge nodes for robust agent performance.

#### 3.6.3 Safety and resource management

- Prevent resource contention by implementing quota enforcement and task prioritization for agent workloads.
- Apply privacy-preserving techniques to safeguard sensitive user data handled by edge-based agents.

### 3.7 Open gateway API ingestion

As agent-based systems evolve within telecom networks, seamless integration with external services is essential. A key enabler of such integration is standardized API access, allowing agents to interact with network functions, cloud platforms, and third-party services. The CAMARA project (CAMARA, 2025), under the Linux Foundation, defines and develops APIs for the GSMA Open Gateway framework (GSMA, 2025), which aims to make telecom networks programmable via open interfaces. Integrating CAMARA APIs into agent-based ecosystems can significantly enhance agent capabilities, enabling real-time data access, dynamic service invocation, and scalable interoperability across heterogeneous systems. This integration should focus on:

#### 3.7.1 API integration for agent interoperability

- Investigating how CAMARA APIs can be used to enable communication between agents and external services, such as network functions, cloud resources, and third-party platforms.
- Defining the protocols for secure, efficient, and reliable API-based interactions within the agent ecosystem.

#### 3.7.2 Enhancing agent capabilities via CAMARA

- Exploring how CAMARA APIs can enable real-time data exchange and provide agents with access to network state information, service exposure, and other vital resources.
- Enabling dynamic agent functionalities by allowing them to interact with a broader range of telecom services via API calls.

#### 3.7.3 Standardization and scalability

- Promoting the adoption of Open Gateway/CAMARA API standards to ensure interoperability across various systems and telecom vendors.
- Ensuring that agents can scale effectively by utilizing the open and standardized nature of CAMARA APIs, allowing seamless integration across 5G and future 6G networks.

#### 3.7.4 Safety considerations

- Implementing strict authentication and access control policies to prevent unauthorized API usage.
- Ensuring API interactions follow rate-limiting and monitoring mechanisms to prevent abuse and potential security breaches.

### 3.8 Multi-agent orchestration and development

In RAN implementations, deploying multiple agents across network layers is essential for optimizing performance, automating operations, and enhancing decision-making. These agents—often based on a shared LLM—must collaborate in real time to manage tasks effectively. However, orchestrating and managing a swarm of such agents introduces new complexities that necessitate standardization. AgentOps, the structured approach to agent development, deployment, and monitoring, is critical for integrating these agents into RANOps—the broader operational framework for RAN automation. Key challenges include autonomous coordination, role assignment, and lifecycle management across heterogeneous agents and network layers. The following outlines the primary standardization areas required to support scalable multi-agent orchestration within telecom environments.

#### 3.8.1 Autonomous multi-agent orchestration

- Designing orchestration frameworks for agents operating at the same RAN layer to coordinate toward shared RANOps objectives.
- Enabling autonomous task distribution, coordination, and conflict resolution to sustain optimal performance, supporting scalable AgentOps workflows.

#### 3.8.2 Life-cycle management

- Defining end-to-end lifecycle processes—from deployment to decommissioning—that align with standardized AgentOps practices.
- Enabling dynamic instantiation, updates, and termination of agents based on real-time network demands and KPIs.

#### 3.8.3 Standardization of agent interaction models

- Establishing standardized interaction protocols that support seamless collaboration across RAN layers under a unified RANOps model.
- Facilitating efficient knowledge, context, and data sharing among agents to enhance swarm intelligence and system-wide coordination.

#### 3.8.4 Scalability and robustness

- Developing methods to scale agent systems across diverse RAN components without degrading performance.
- Ensuring resilience by enabling agents to self-heal, reconfigure, or adapt autonomously in response to network failures or changes.

#### 3.8.5 Safety considerations

- Introducing conflict mitigation and behavioral constraints to prevent unsafe emergent behavior in multi-agent environments.
- Embedding security and anomaly detection into lifecycle management to safeguard AgentOps operations from adversarial threats.

### 3.9 Reasoning and resilience frameworks for RAN agentics

To enable robust and intelligent behavior within GenAI-driven RAN architectures, agents must be equipped with standardized reasoning frameworks that combine classical context-aware methods with modern generative techniques. Traditional approaches rooted in information and communication theory (Glover and Grant, 2009; Shannon, 1948) have long supported effective decision-making in telecom systems. Integrating these with GenAI enhances the adaptability and robustness of agents, supporting more dynamic and resilient RAN operations.

#### 3.9.1 Combining classical reasoning with GenAI

- Explore hybrid models that merge classical signal processing and information-theoretic reasoning with GenAI capabilities to improve agent decision-making in complex, real-time environments.
- Enable agents to leverage structured rules alongside generative inference, improving their capacity for autonomous orchestration and adaptation across RAN layers.

#### 3.9.2 Leveraging statistical abstractions

- Prioritize the use of statistical abstractions over raw time-series data to reduce complexity and enhance interpretability in planning and tool invocation.
- Identify standardized methods for integrating statistical insights into agent workflows, enabling more efficient and consistent performance across deployments.

#### 3.9.3 Incorporating domain-specific processing

- Investigate domain-specific techniques—such as frequency-domain or delay-Doppler processing—as standardized modules for enhancing agents' understanding of network behavior.
- Use these specialized methods to support more precise and context-aware agent decisions, especially in performance-sensitive RAN scenarios.

#### 3.9.4 Safety and resilience mechanisms

- Establish verification protocols and fallback strategies to mitigate the risks of faulty reasoning or hallucinated outputs within autonomous agents.
- Integrate safety mechanisms into the agent development lifecycle to ensure resilience under varying network conditions and failure scenarios.

## 4 Conclusion

The urgency for standardization arises from the increasing complexity of RAN environments, the growing reliance on AI-driven decision-making, and the need for seamless coordination between AI agents and traditional network functions. Without clear guidelines, the adoption of GenAI in RAN poses safety and security risks, along with fragmentation, inefficiencies, and inconsistencies in implementation. The standardization of agentic architectures, fine-tuning methodologies, benchmarking frameworks, and AI-friendly documentation is essential to ensure scalability, reliability, and compliance with existing telecom regulations. Future efforts should focus on refining the interoperability and robustness of these systems, ensuring that they can effectively support the evolving demands of 5G and 6G networks. Collaborative efforts within the telecom industry will be crucial to overcoming these challenges, making AI-driven automation a key enabler of next-generation network operations.

## Data Availability

The original contributions presented in the study are included in the article/supplementary material, further inquiries can be directed to the corresponding author.

## Footnotes

^1^NVIDIA on Telecom Agentic AI, https://blogs.nvidia.com/blog/telecom-agentic-ai-for-network-operations/, accessed Mar. 2025.
